# Ultrasonic-Assisted Decoloration of Polysaccharides from Seedless Chestnut Rose (*Rosa sterilis*) Fruit: Insight into the Impact of Different Macroporous Resins on Its Structural Characterization and In Vitro Hypoglycemic Activity

**DOI:** 10.3390/foods13091349

**Published:** 2024-04-27

**Authors:** Guangjing Chen, Meiwen Sun, Kaiwen Chen, Lisha Wang, Juyan Sun

**Affiliations:** 1 College of Food Science and Engineering, Guiyang University, Guiyang 550005, China; 15286718590@163.com (M.S.); chenkaiwen2001@126.com (K.C.); 17585419235@126.com (J.S.); 2 College of Life Sciences, South China Agricultural University, Guangzhou 510642, China; 3 Experimental Center, Guizhou Police College, Guiyang 550005, China; gzpcwls1989@126.com

**Keywords:** seedless chestnut rose, polysaccharides, decoloration, ultrasonication, macroporous adsorbent resin, structure, *α*-glucosidase

## Abstract

Pigments within polysaccharides pose significant challenges when analyzing their structural characteristics and evaluating their biological activities, making decolorization a crucial step in purifying these biomolecules. In this research, a novel approach using ultrasound-assisted static adsorption with macroporous resins was employed to decolorize polysaccharides extracted from seedless chestnut rose (*Rosa sterilis* S. D. Shi) fruit (RSP). Among the fourteen tested resins, AB-8, D101, D4020, HPD100, and S8 were identified as the most effective, demonstrating superior decoloration efficiency and polysaccharide recovery. Further examinations of RSPs treated with these five resins revealed distinct effects on their uronic acid levels, monosaccharide makeup, molecular weight, surface structure, and hypoglycemic properties. The RSP treated with HPD100 resin stood out for having the highest uronic acid content, smallest particle size, and lowest molecular weight, leading to the most notable inhibition of α-glucosidase activity through a mixed inhibition model. The application of HPD100 resin in the decolorization process not only potentially preserved the macromolecular structure of RSP but also enhanced its hypoglycemic efficacy. These findings provide a solid theoretical basis for further exploring RSP as a component of functional foods, underscoring the effectiveness of the ultrasound-assisted resin adsorption method in polysaccharide purification.

## 1. Introduction

Polysaccharides are extensive natural polymers with a diverse array of biological functions. Research has highlighted their significant potential in promoting immune health [1], providing cardiovascular protection [2], regulating gut microbiota [3], and managing metabolic disorders [4]. Over recent years, the appeal of plant-based polysaccharides has surged in the realms of food, pharmaceuticals, and biomedicine, attributed to their hydrophilic nature, stability, safety, non-toxicity, and biodegradability, positioning them as sustainable and environmentally friendly resources [5]. However, the extraction and preparation of polysaccharides often encounter a significant challenge due to the presence of pigments in the natural plant materials, such as roots, stems, leaves, flowers, and fruits. These coexisting pigments can complicate the processes of separation, purification, and structural characterization, potentially impacting the biological activity of the polysaccharides [6,7]. Specifically, pigments may interfere with the experimental outcomes, particularly in assays related to colorimetric analyses such as protein, total sugar, and reducing sugar determinations, as well as in structure and activity analyses [8]. Moreover, pigments could influence the aesthetic appeal and consumer perception of product color, which is critical in food products like baby formulas, dairy products, beverages, and confectioneries [9]. Given these considerations, decolorization is recognized as a critical step in the preparation process of plant polysaccharides, ensuring their purity and efficacy for industrial application.

**Figure 1:**
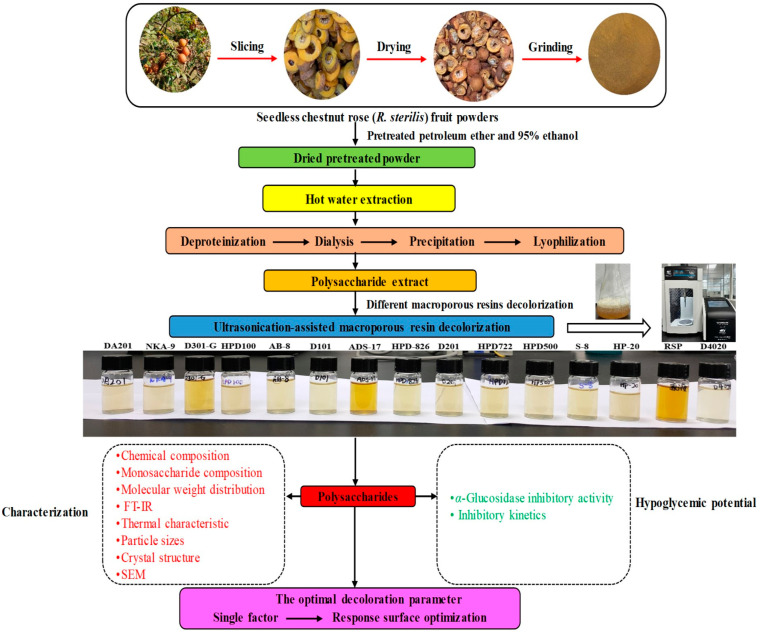
The schematic of the experimental design.

Golden Cili, also known as seedless chestnut rose (*Rosa sterilis* S. D. Shi, RS), is a distinctive perennial vine native to the Guizhou Province, China. Identified as a new *R. roxburghii* Tratt (RRT) variety by the Guizhou Botanical Garden over thirty years ago, RS is known for its attractive seedless and vitamin-C-rich fruits [10]. Thriving in the mountainous terrains of Anshun, Guizhou, RS shares significant genetic similarities with RRT. Past studies extensively investigated RRT’s beneficial properties, including high levels of polysaccharides, ascorbic acid, superoxide dismutase and phenolic compounds. These properties suggest potential applications in preventing atherosclerosis, inhibiting cancer cell growth, and treating type 2 diabetes [11,12]. Conversely, studies on RS, especially regarding its polysaccharides, remain limited. Existing research has mainly focused on small molecular compounds in RS fruit, such as flavonoids, phenols, terpenoids, and steroids [13,14,15,16]. Remarkably, RS fruits are rich in polysaccharides. Oue preliminary experiments have not only successfully extracted these polysaccharides from the fruit, achieving a yield of 7.11%, but have also uncovered their potent hypoglycemic effects [17]. However, the extraction process yielded a crude RSP laden with dark yellow pigments (Figure 1), presenting significant challenges for its application in the food industry and beyond due to these pigment impurities. Moreover, these impurities posed a risk of contaminating DEAE-52 cellulose and Sephacryl S-400 HR columns used in the further separation and purification of RSP, thus escalating operational costs and complicating the analysis of its structure and functionality. Thus, addressing the decolorization of RSP emerges as a critical issue requiring immediate attention.

Currently, the decolorization of polysaccharides is primarily attained through the following three methods: oxidation with hydrogen peroxide (H_2_O_2_), activated carbon adsorption and macroporous resin adsorption [8]. H_2_O_2_ can achieve decolorization by its strong oxidation ability, but this power can also change the structure and functional groups of polysaccharides, reducing their effectiveness in biological processes [18]. On the other hand, activated carbon adsorption, which relies on van der Waals and electrostatic forces to capture pigments, suffers from poor selectivity between pigments and polysaccharides [19]. This lack of specificity often leads to substantial polysaccharide loss and challenges in separating the activated carbon, rendering the process inefficient and time-consuming [20]. In contrast, macroporous adsorption resin stands out for its stability, high efficiency, cost-effectiveness, resin recyclability, and ease of operation, making it a preferred method for polysaccharide decolorization [6]. Macroporous resins like AB-8, ADS-7, D101, D280, D380, D941, S-8, X-5, and NKA-II have been extensively employed and proven to be effective in the decolorization of polysaccharides, demonstrating their viability as a superior method in industrial applications [6,7,21,22,23].

Macroporous resins are specialized organic polymers, synthesized through polycondensation or polymerization, characterized by their expansive pore network and significant surface area. These resins operate through the formation of physical and/or chemical bonds, adsorbing compound molecules onto their surface [24]. Available in diverse forms such as hydrophobic, hydrophilic, ionic, and non-ionic, their selection is tailored to the specific physicochemical properties of the target compound. The adsorption capabilities of these resins depend on mechanisms such as surface adsorption dynamics, sieve effects, electrostatic properties, and hydrogen bonding [22]. To enhance their efficiency, macroporous resins may undergo treatments like heating, ultrasonication, or chemical modification, changing their physical and/or chemical traits. Particularly, ultrasonication emerges as an innovative approach, improving resin adsorption capacity through its mechanical and cavitational effects, which boosts mass transfer between the solution and adsorbent [25]. This technique has shown promise in increasing the adsorption of various substances, including organic contaminants and certain bioactive compounds like anthocyanins, polyphenols, and flavonoids [24,26,27,28]. However, the application of ultrasound-assisted static macroporous resin adsorption (USMRA) for polysaccharide decolorization, particularly regarding RSP, remains unexplored. Current research primarily focuses on the physicochemical differences in polysaccharides pre- and post-decolorization, lacking comprehensive analyses on how decolorization affects their structural and functional integrity [22,23,29]. Consequently, there is a gap in understanding the structure–activity relationship of polysaccharides pre- and post-decolorization. Furthermore, considering the variety of available resins, selecting the appropriate resin based on the specific physicochemical attributes of the polysaccharide is crucial for achieving optimal decolorization outcomes.

Accordingly, in this study, an efficient approach was devised for the decolorization of RSP using an optimized USMRA technique. The process began with screening fourteen macroporous resins, each varying in chemical and physical properties, to identify the optimum resin based on decolorization efficiency, RSP recovery ratio, and selectivity coefficient. Subsequently, the influences of different macroporous resins on the structure and in vitro α-glucosidase inhibitory effects of RSP were evaluated to elucidate the association between the structure and the hypoglycemic activity. A schematic of the experimental process is outlined in Figure 1.

## 2. Materials and Methods

### 2.1. Materials and Reagents

The RS fruits were obtained from Xixiu, Anshun, Guizhou Province, China (106°6′57.22″ E, 26°8′55.17″ N, 1268.3 m above sea level), in October 2021. TOKYO Chemical Industry Co., Ltd., Tokyo, Japan, provided 3-phenylphenol, and trifluoroacetic acid (TFA) and standard monosaccharides. Thermo Fisher Co., Waltham, MA, USA, supplied sodium hydroxide, while the pullulan was procured from Agilent Co., Santa Clara, CA, USA. Yuanye Bio-technology Co., Ltd., Shanghai, China, provided serum albumin (BSA), *p*-nitrophenyl-α-d-glucopyranoside (PNPG), and α-glucosidase.

### 2.2. Macroporous Resins and Pretreatment

Fourteen macroporous resins, namely AB-8, ADS-17, D101, D201, D301-G, D4020, DA201, HP-20, HPD100, HPD500, HPD722, HPD826, NKA-9, and S-8, were supplied by Yuanye Bio-technology Co., Ltd., Shanghai, China. The physicochemical characteristics of these resins are detailed in Table 1. Prior to the adsorption studies, the resins underwent a modified pretreatment process as advised by the supplier. Initially, to eliminate surface impurities, pre-measured quantities of resins were rinsed with distilled water. Subsequently, they were immersed in 95% ethanol for 24 h to remove ethanol-soluble substances. Post-swelling, the resins were washed with distilled water to remove any ethanol residues. To remove monomers and porogenic agents trapped within the pores post-synthesis, the resins underwent sequential treatments, initially with a 5% hydrochloric acid (HCl) solution, followed by a 3% sodium hydroxide (NaOH) solution. Subsequently, the resins were rinsed repeatedly with distilled water until achieving a neutral pH, after which they were preserved at 4 °C for subsequent application.

### 2.3. Preparation of Crude RSP Extract

The process began with slicing whole RS fruits, followed by oven-drying at 55 °C for 48 h, and with a 60-mesh sieve, grinding into a fine powder. The powder was then decolorized and degreased three times with 95% ethanol (*v*/*v*) and petroleum ether. After airing to remove residual solvent, the powder was oven-dried 24 h at 40 °C, and stored at −80 °C. After hot water extraction and alcoholic precipitation, the crude RSP was obtained. The powder was then mixed with distilled water (1:20 ratio) and heated for 2 h at 90 °C, followed by cooling and 10-min centrifugation at 4000 rpm, and the precipitate was separated for a second extraction. The combined supernatants were vacuum-concentrated at 53 °C to a one-third volume. The Sevag method was used to remove proteins [30], and the resulting solution was dialyzed (molecular weight cutoff: 8000–14,000 Da) for two days against distilled water. Four volumes of absolute ethanol were then added, and after overnight incubation at 4 °C, the sample was centrifugated for 10 min at 4000 rpm, and the obtained sediment was freeze-dried.

### 2.4. Preliminary Screening of Resins Using USMRA

**Figure 2:**
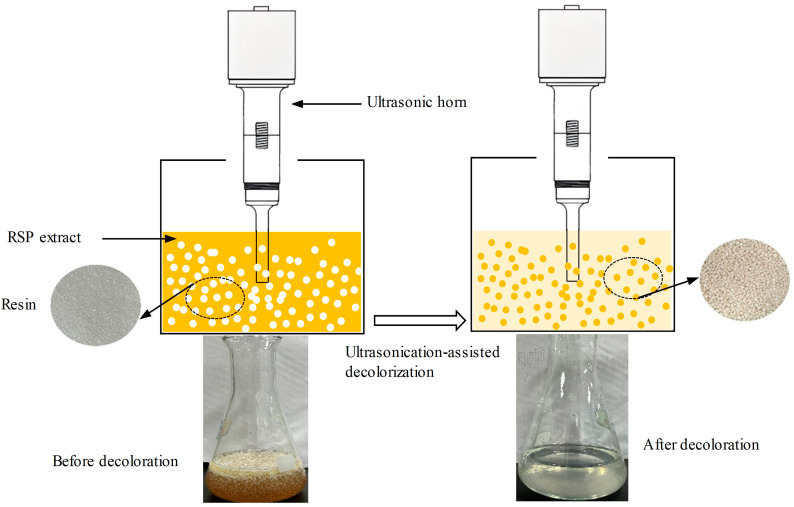
Diagram of ultrasound-assisted static macroporous resin adsorption for RSP decolorization.

To identify the optimal macroporous adsorption resins, an evaluation of fourteen distinct types was conducted utilizing the USMRA technique. In this process, 20 g of each pre-treated resin was introduced into 100 mL of the crude RSP solution (concentration: 2.5 mg/mL in distilled water). This mixture was then subjected to sonication at 400 W using a 10 mm ultrasonic probe (JY92-IIDN, Ningbo Scientz Biotechnology Co., Ltd., China) for 70 min at a controlled temperature of 40 °C, facilitated by a temperature-controlled bath (Figure 2). Subsequent to the adsorption phase, the resin was isolated from the mixture through a filtration process and then retrieved. The selection of suitable resins was based on the following three critical parameters: the decoloration ratio (*D_r_*), polysaccharide recovery ratio (*R_r_*), and selectivity coefficient (*K_c_*). The quantification of polysaccharide levels was executed via the phenol-sulfuric acid method, using glucose as a calibration standard [31]. The *R_r_* was ascertained by measuring absorbance at 420 nm with a UV–visible spectrophotometer [7]. The *K_c_* was determined following established methods, as detailed in the formulas provided [22]:

(1) $$D_{r}\;\left(\%\right)=\;(A_{D0}-A_{D1})\;/A_{D0}\times 100$$

(2) $$R_{r}\;\left(\%\right)=A_{R1}\;/A_{R0}\times 100$$

(3) $$K_{d1}=\frac{(A_{D0}-A_{D1})\;/M}{A_{D1}}$$

(4) $$K_{d2}=\frac{(A_{R0}-A_{R1})\;/M}{A_{R1}}$$

(5) $$K_{c}=K_{d1}\;/K_{d2}$$

where *A_D_*_0_ and *A_D_*_1_ represent the initial and final absorbance measurements of the RSP solution at a wavelength of 420 nm, before and after the decolorization process, with *D_r_* indicating the decolorization ratio; *A_R_*_0_ and *A_R_*_1_ denote the absorbance measurements at 490 nm before and after decolorization, following coloration with sulfuric acid phenol reagent, with *Rr* reflecting the polysaccharide recovery ratio. The terms *K_d_*_1_ and *K_d_*_2_ refer to the equilibrium distribution coefficients for the pigments and RSP within the resin matrix, while *K_c_* stands for the selectivity coefficient among them; *M* signifies the resin’s mass in grams.

### 2.5. Chemical Composition of RSP before and after Decoloration with Different Macroporous Resins

The determination of total sugar levels was carried out employing the phenol-sulfuric acid method, with glucose serving as the standard [31]. The content of uronic acid was quantified utilizing the *m*-hydroxybiphenyl approach, with galacturonic acid acting as the reference [32]. Meanwhile, the quantification of protein levels was conducted through Bradford’s method, where bovine serum albumin was used as the calibration standard [33].

The composition of monosaccharides in each RSP was scrutinized using high-performance anion exchange chromatography as outlined in previously established methods [34]. RSP samples in sealed tubes were hydrolyzed with 3 M TFA at 120 °C for 3 h. Subsequently, to remove TFA, the hydrolyzed samples were subjected to triple evaporation under rotary vacuum conditions, coupled with methanol co-distillation, to remove TFA. After reconstituting the hydrolysates or sugar standards in distilled water, they underwent filtration through a 0.22 μm membrane. The system (ICS-6000, Thermo Scientific Co., Waltham, MA, USA) was utilized for analysis, featuring a Dionex CarboPac^TM^ PA20 analytical column (3 mm × 150 mm), and a Dionex CarboPac^TM^ PA20 safeguard column (3 mm × 30 mm) at a flow rate of 0.3 mL/min. The chromatographic separation was achieved using a mobile phase consisting of the following three solutions: 15 mM NaOH (A), 0.25 M NaOH (B), and 15 mM NaOH with 0.1 M NaAc (C), at a flow rate of 0.3 mL/min. The gradient elution was programmed to start with 100% solution A (15 mM NaOH) for the first 25 min, switched to 100% solution C (15 mM NaOH with 0.1 M NaAc) for the next 27 min, and conclude with 100% solution B (0.25 M NaOH) from 52.1 to 70 min, all at a column temperature of 30 °C. Monosaccharides were identified and quantified based on their elution times, compared against a set of sugar standards, with the monosaccharide concentration expressed as a mole percentage of the total monosaccharides detected.

### 2.6. Structural Characterization of RSP before and after Decoloration with Different Macroporous Resins

#### 2.6.1. Molecular Weight Distribution Determination

High-performance gel permeation chromatography (HPGPC) was utilized for the determination of RSP’s molecular weights with 3 mg of each RSP sample used. Separation utilized two Agilent PL aquagel-OH gel columns (60 and 50 series, 25 mm × 300 mm, 8 μm) with KH_2_PO_4_ (0.02 M, pH 6.0) at 0.6 mL/min, 30 °C, and a 20 μL injection volume. Calibration involved seven pullulan polysaccharide standards (667 Da to 739,000 Da). GPC software (version A.02.02, Agilent Co., Waldbronn, Germany) was used to determine the molecular weight distribution, including the number-average (Mn) and weight-average (Mw) molecular weights and the polydispersity index (Mw/Mn).

#### 2.6.2. Fourier Transform Infrared (FT-IR) Spectra

FT-IR spectra were acquired using a Bruker-VERTEX 70 FT-IR spectrometer (Ettlingen, Germany). For sample preparation, about 1.0 milligrams of the polysaccharide were completely blended with 200 milligrams of high-quality KBr for spectroscopy and compacted into a tablet. The examination was carried out at intervals of 4 cm^−1^ over a range of wavenumbers from 4000 to 450 cm^−1^.

#### 2.6.3. Particle Size

A homogeneous solution of RSPs was prepared at a concentration of 0.50 mg/mL using distilled water. The determination of the particle size of RSPs was conducted with a Zetasizer Nano-ZS90 (Malvern, UK), with the measurements recorded at a temperature of 25 °C.

#### 2.6.4. X-ray Diffraction (XRD)

XRD analysis was employed to evaluate the structural characteristics of RSPs, focusing on their crystalline or amorphous attributes. The D8 Advance diffractometer with a copper anode for Cu Kα radiation was used to capture XRD spectra. Instrument parameters were set to 40 kV and 40 mA for voltage and current, respectively. Data collection ranged from 5 to 80° in 2θ, with a step size of 0.02° and a dwell time of 1 s/step.

#### 2.6.5. Thermal Stability Properties

RSP samples were assessed via thermogravimetry (TG) and differential thermogravimetry (DTG) using a DTG-60A instrument (Shimadzu, Tokyo, Japan). In this study, samples weighing between 3 and 5 milligrams were exposed to controlled heating in a nitrogen environment, with nitrogen gas flowing at a rate of 50 mL/min. The samples were heated uniformly from 25 °C to 800 °C with a constant increase in temperature at a rate of 10 °C/min.

#### 2.6.6. Scanning Electron Microscopy (SEM)

The examination of the surface structures of different RSP samples was conducted using a SIGMA 300 SEM (ZEISS Group, Oberkochen, Germany), operating at an acceleration voltage of 5.0 kV. To improve electrical conductivity and thus the quality of the images, each sample underwent a gold sputtering process before analysis. Prior to observation, each sample was coated with gold to enhance conductivity. Images were captured at magnifications of 100× and 500×.

### 2.7. α-Glucosidase Inhibition Assays of RSP before and after Decoloration with Different Macroporous Resins

#### 2.7.1. In Vitro α-Glycosidase Inhibitory Activity Assay

The α-glucosidase inhibitory activity was assessed according to established procedures [17]. Different concentrations of RSP solution (50 μL) were mixed with α-glucosidase enzyme solution (50 μL, 0.5 U/mL) and incubated at 37 °C for 10 min. Subsequently, PNPG substrate (50 μL, 5 mM) was added, and the mixture was further incubated at 37 °C for 20 min. The reaction was terminated by adding 200 μL of 1 M Na_2_CO_3_. α-Glucosidase inhibitory activity was determined by measuring the release of *p*-nitrophenol from the PNPG substrate at 405 nm. The effect of different concentrations of RSP on α-glucosidase was assessed by measuring absorbance at 405 nm and calculating using the following formula:

(6) $$Inhibitory\;activity\;\left(\%\right)=\left[1-\left(A_{sample}-A_{blank}\right)/A_{control}\right]\times 100$$

Here, *A_sample_* refers to the absorbance measured from the reaction mixture that includes both RSP samples and the α-glucosidase enzyme; *A_blank_* represents the absorbance when the RSP samples are tested in the absence of α-glucosidase to account for any background absorption by the samples themselves; *A*_*control*_ denotes the absorbance of the control reaction without any RSP samples, serving as a baseline for maximum enzyme activity; To quantify the effectiveness of RSPs, their half maximal inhibitory concentration (IC_50_) was calculated according to the non-linear regression analysis. The IC_50_ value indicates the concentration of RSPs required to achieve 50% inhibition of α-glucosidase activity in vitro.

#### 2.7.2. Kinetic Characterization of Inhibition

The kinetic models for the inhibition of α-glucosidase by RSPs were established based on concentrations of PNPG (1.0, 2.0, 3.0, 4.0 mM) and RSPs (0, 2.0, 4.0 mg/mL), following methodologies outlined in previous research [34]. The initial reaction velocity (*v*) was determined by the change in absorbance during the first 5 min of the reaction, which reflects the early phase of α-glucosidase activity on PNPG. To elucidate the type and mechanism of inhibition that RSPs impose on α-glucosidase, Lineweaver–Burk plots were constructed. This approach involves plotting the reciprocal of the reaction velocity (1/*v*) against the reciprocal of the substrate concentration (1/[*S*]) in a linear regression analysis. These plots facilitate the determination of key enzymatic parameters, including the Michaelis–Menten constant (*K_m_*) and the maximal reaction velocity (*v*_max_), in accordance with the Michaelis–Menten equation [35], which is as follows:

(7) $$\frac{1}{v}=\frac{1}{v_{\max}}+\frac{K_{m}}{v_{\max}}\cdot \frac{1}{\left[S\right]}$$

Specifically, the relationship between the 1/*v* and the concentrations of the inhibitor [*I*] and substrate [*S*] can be elegantly depicted using the Cornish-Bowden–Eisenthal plot [36], as follows:

(8) $$\frac{1}{v}=\frac{1}{v_{\max}}\cdot (1+\frac{\left[I\right]}{K_{ii}})+\frac{K_{m}}{v_{\max}}\cdot (1+\frac{\left[I\right]}{K_{i}})\cdot \frac{1}{\left[S\right]}$$

where *K_i_* is defined as the dissociation constant that quantifies the separation of the inhibitor (*I*) and the enzyme (*E*) from their combined form (EI). Concurrently, *K_ii_* represents the inhibition constant, which measures the transformation efficiency of the enzyme–substrate–inhibitor (ESI) complex back into the enzyme–substrate (ES) complex.

### 2.8. Statistical Analysis

Data were reported as mean ± standard deviation (SD). The collection of data samples was facilitated using Excel 2021 software (Microsoft, Redmond, WA, USA). These samples were then graphically represented with the assistance of OriginPro 2021 Learning Edition software (Origin Lab Corp., Northampton, MA, USA) and underwent analysis via IBM SPSS Statistics 27.0 software (SPSS Inc., Chicago, IL, USA). Statistical significance was determined using Duncan’s test, with a threshold of *p* < 0.05 indicating significance.

## 3. Results and Discussion

### 3.1. Selection of Optimal Decoloration Resin

**Figure 3:**
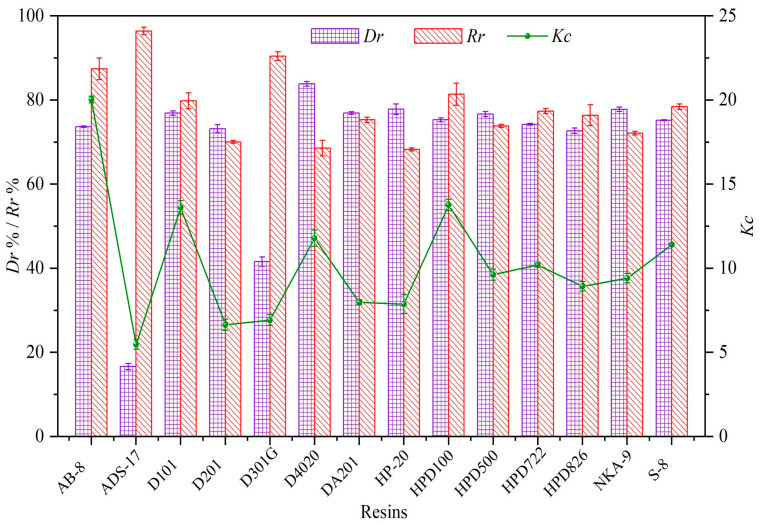
Comparison of decoloration ratios (*D_r_*%), RSP recovery ratios (*R_r_*%) and selectivity coefficients (*K_c_*) of different resins.

The effectiveness of resin adsorption is influenced by several factors, including surface adsorption, molecular sieving, surface charge properties, and hydrogen bonding interactions [37]. When a liquid phase moves by a solid adsorbent, substances in the fluid bond with the particles and compounds located on both the external surface and within the internal cavities of the resin [38]. The outer layer of a macroporous resin is recognized for possessing specific electronic and spatial characteristics, leading to an energetically diverse environment. Simultaneously, the interior porous layer plays a pivotal role in enhancing the adsorption process [39]. Consequently, the decolorization effect is closely linked to both the external and internal surface areas, pore sizes, and functional groups of the resin. For effective decolorization, resin should exhibit high efficiency in pigment adsorption and the ability to recover polysaccharides. In this study, 14 macroporous resins were evaluated for their pigment adsorption capabilities. These included four non-polar resins (D101, D4020, HP-20, and HPD100), three weakly polar non-ionic resins (AB-8, D301-G, and HPD722), two resins capable of hydrogen bonding (ADS-17 and HPD826), and five strongly basic anionic exchange resins (D201, DA201, HPD500, NKA-9, and S-8). The selection criteria for the optimal resin for decolorizing RSP included *D_r_*, *R_r_* and *K_c_*. Analysis revealed that, apart from ADS-17 and D301G, all resins demonstrated decolorization rates exceeding 70%, showcasing their substantial ability to adsorb pigment impurities from RSP (Figure 3). Moreover, AB-8 and HPD100 resins were the only ones that had decolorization rates above 70% and recovery rates above 80%, highlighting their superior performance. After evaluating the decolorization efficiency, RSP recovery rates, and selectivity coefficient, AB-8, D101, D4040, HPD100 and S8 resins were selected for subsequent experiments.

### 3.2. Changes in Chemical Compositions after Being Treated by Five Resins

After applying a decolorization process using USMRA on five different resins, we assessed the impact on the sugar, protein, and uronic acid levels in both the original RSP and decolored RSP. As outlined in Table 2, carbohydrates emerged as the dominant component of RSP, evidenced by a total sugar content of 61.80 ± 2.62%. The protein content was minimal, consistent with previous findings [34]. Noteworthy variances between the original and treated RSP were primarily observed in their sugar and uronic acid quantities (*p* < 0.05), with the treated RSP displaying a marked increase in these components compared to its original state (*p* < 0.05). Among the various treated samples, RSR-D4020 showed the highest sugar concentration, whereas RSP-HPD100 exhibited the highest uronic acid level. These findings underscored that decolorization not only elevated the purity and uronic acid levels of RSP but also that these enhancements were influenced by the specific macroporous resin employed.

### 3.3. Changes in Structure Characterizations after Being Treated by Five Resins

#### 3.3.1. Monosaccharide Composition

The composition and ratio of monosaccharides in RSPs, as depicted in Figure S1 and Table 2, demonstrate a consistent makeup across all samples, including Fuc, Rha, Ara, Gal, Glc, xylose Xyl, Man, and GalA. Although the type of monosaccharides remained unchanged by various decolorization macroporous resins, significant alterations were noted in the concentrations of Gal, Glc and GalA. Notably, the GalA levels in the decolored RSP samples were significantly elevated when compared to the untreated RSP. The high GalA content in RSP indicates its primary composition as a pectic polysaccharide. This implied a potential for high α-glucosidase inhibitory activity, in line with prior research linking high uronic acid levels to biological efficacy [40]. Particularly, the GalA concentrations in the decolored samples RSP-D101 (35.28%) and RSP-HPD100 (35.19%) were significantly higher than in the original RSP (20.45%), hinting at enhanced bioactivity following decolorization. Furthermore, the variance in Gal, Glc and GalA content across different RSP types implied that the ultrasound-assisted decolorization process could impact the structural conformation of polysaccharides.

#### 3.3.2. Molecular Weight Distribution

**Figure 4:**
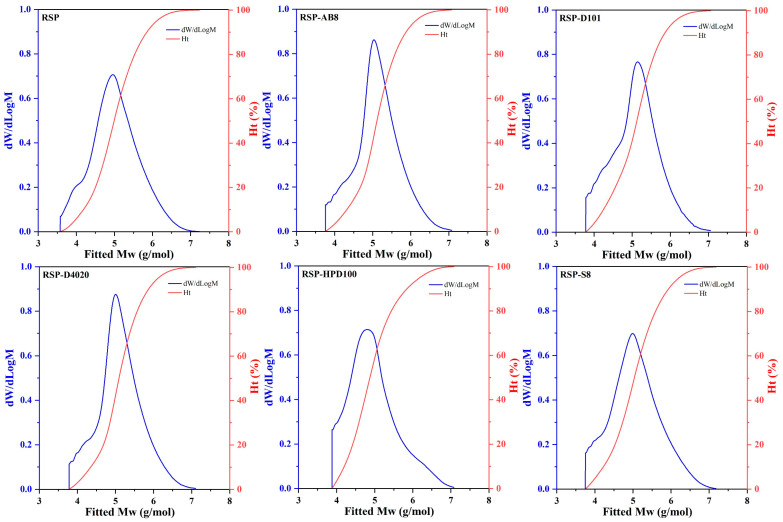
The molecular weight distribution curves of RSP before and after decoloration with different macroporous resins.

The molecular weight distribution changes between the original RSP and its decolored variants were analyzed using HPGPC. This analysis considered the Mn, Mw, and the polydispersity index (Mw/Mn) to evaluate the homogeneity of RSP samples. As shown in Figure 4, all samples exhibited a singular distribution curve, reflecting their uniformity. According to data presented in Table 2, the native RSP possessed an Mw of 307.54 kDa. Upon treatment with USMRA, significant increases in Mw were noted for the RSP-AB8, RSP-D101, and RSP-S8 samples, unlike the RSR-D4020 and RSP-HPD100 samples, which showed no substantial alterations. This pattern revealed a tendency for polysaccharide molecules to aggregate when treated with AB8, D101, and S8 resins. Polysaccharides exhibiting lower polydispersity indices, reflecting a tighter molecular weight distribution, are considered more homogeneous. The original RSP’s polydispersity index was 7.56, indicating a wide molecular weight range and thus a lower level of homogeneity. After decolorization treatment, the indices for RSP-AB8, RSP-D101, and RSR-D4020 notably decreased, whereas RSP-HPD100 and RSP-S8 experienced negligible shifts. This decrease for the former samples implied these resins caused RSP aggregation and trapped smaller molecular weight fractions during the decolorization process. These observations indicated that the molecular weight distribution of RSP is influenced by the type of resin used in the decolorization process. Among the five resins examined, AB8, D101, D4020, and S8 altered the molecular weight profile of RSP, whereas HPD100 maintained it.

#### 3.3.3. FT-IR Spectra Analysis

**Figure 5:**
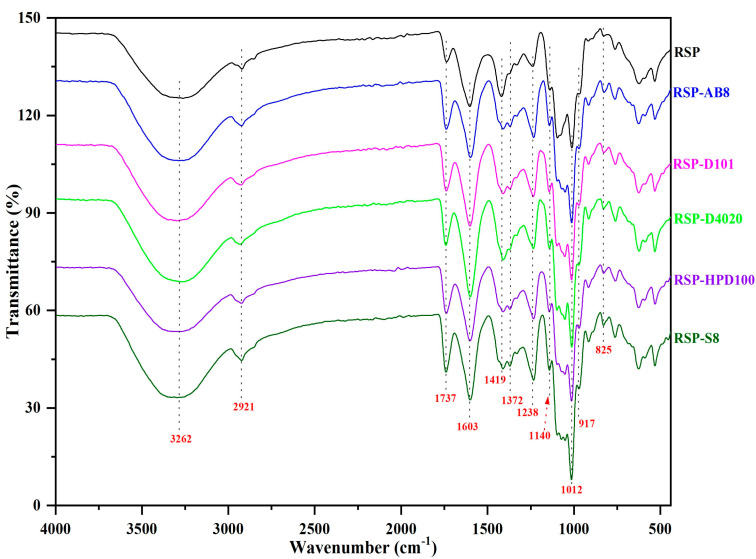
The FT-IR spectra of RSP before and after decoloration with different macroporous resins.

The FT-IR spectroscopy results for both the original and decolored RSP samples are displayed in Figure 5. Across all samples, there was a consistent observation of similar absorption peaks, which included the stretching vibrations of hydroxyl groups (–OH) at 3262 cm^−1^, the stretching vibrations of C–H at 2921 cm^−1^, the stretching vibrations of the esterified carboxylic groups at 1737 cm^−1^, the stretching vibrations of free carboxyl groups at 1603 cm^−1^, and the C–O bonds tensile vibrations at 1419 cm^−1^ [41]. Additionally, the presence of pyran rings was confirmed through the symmetric vibrations of C–O–C or C–O–H bonds, resulting in absorption peaks from 1140 cm^−1^ to 1012 cm^−1^ [42]. The two bands at 917 cm^−1^ and 825 cm^−1^ signaled the existence of β- and α-configurations of sugar molecules [43]. A notable distinction was observed in the intensity of the FT-IR absorption bands at 1603 cm^−1^ among the samples. The decolored RSP samples exhibited a stronger absorption peak at this wavelength compared to the original RSP, indicative of an increased uronic acid content in the decolored samples. The outcomes supported the uronic acid content and monosaccharide composition analysis results, confirming the efficacy of decolorization assisted with ultrasound utilizing different resins. Despite shifts in uronic acid levels and monosaccharide composition, the fundamental structural elements, like glycosidic bonds and sugar rings, remained unaltered during the decolorization procedure.

#### 3.3.4. The Average Particle Size and Crystal Structure

**Figure 6:**
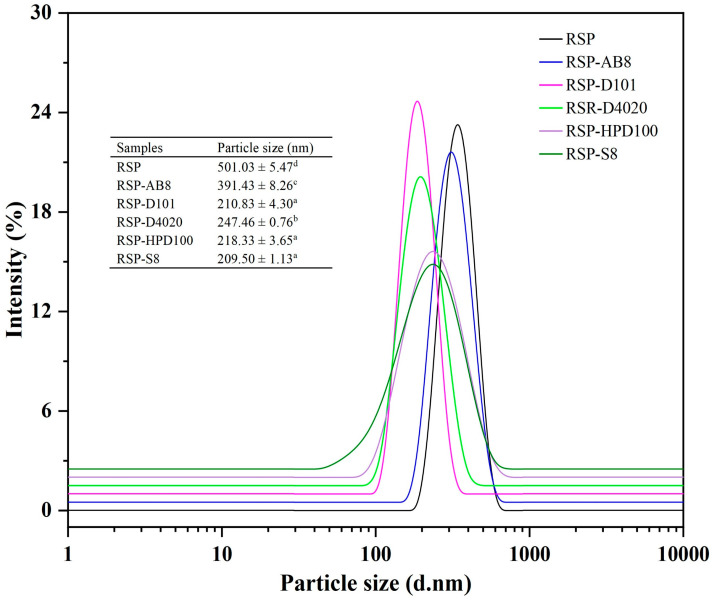
The particle size distribution curves of RSPs before and after decoloration with different macroporous resins. Values for particle size with no letters in common are significantly different (*p* < 0.05).

The stability of a solution can frequently be deduced from examining the hydrodynamic properties of its constituent particles. The average particle sizes of both the untreated and decolorized RSP samples were analyzed, with the results illustrated in Figure 6. The data revealed that all RSP samples exhibited broad and even size distributions. Notably, after undergoing USMRA process, the average size of RSP particles was significantly reduced from 501.03 nm to 209.50 nm. Among these, the RSP-D101 sample stood out for its notably narrower and more uniform particle size distribution, indicating a higher stability in aqueous solutions compared to other decolored RSP variants. X-ray diffraction (XRD) analysis serves as a pivotal technique for assessing the phase and crystalline structure of materials. Despite both the original and decolored RSP samples displaying an amorphous nature as verified by XRD examination (Figure S2), a discernible decrease in diffraction peak intensities was observed in the decolored samples, indicating a slight reduction in crystallinity post-USMRA treatment. This reduction in crystallinity could be attributed to the ultrasonic treatment disrupting the organization of the polysaccharide chains, thereby diminishing their overall crystalline structure.

#### 3.3.5. Thermal Analysis

Thermogravimetric analysis (TG) is a crucial technique for assessing the thermal stability and compositional attributes of polysaccharides. The TG and derivative thermogravimetry (DTG) profiles of six RSP polysaccharides samples are displayed in Figure S3, revealing three primary phases of mass loss upon heating, as detailed in Table 3. The initial phase of mass loss was associated with the evaporation of absorbed and bound water, alongside other volatile substances within the RSP samples [44]. The variation in water loss across these samples highlighted differences in their water content. The subsequent phase was characterized by the degradation of glycosidic bonds and the disassembly of polysaccharide side chains [45]. In the final decomposition stage, more resilient structures like glucosidic groups and C–O bonds broke down, ultimately leading to the degradation of the polysaccharide backbone [46]. The half-life temperatures (*T*_50_), the point at which the mass of each sample was halved, were recorded for each RSP, as follows: RSP, RSP-AB8, RSP-D101, RSP-D4020, RSP-HPD100, and RSP-S8, with values of 296.3, 295.9, 295.1, 294.9, 295.0, and 296.7 °C, respectively (*p* > 0.05). Additionally, the total mass losses among these samples were not significantly different, indicating that the ultrasound-assisted decolorization process did not substantially affect the thermal stability of RSP.

#### 3.3.6. SEM

**Figure 7:**
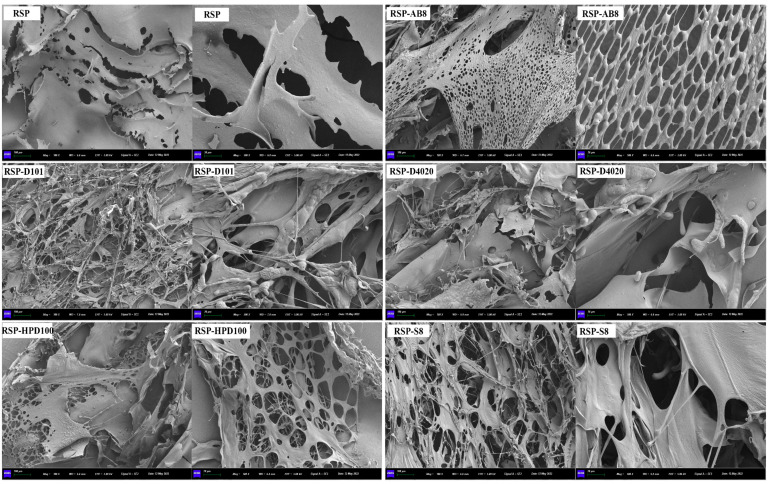
The surface morphology of RSP before and after decoloration with different macroporous resins.

The surface morphology of both the original and decolored RSP samples, as visualized under 100× and 500× magnification, is presented in Figure 7. Each of the RSP displayed distinctive differences in size, shape, and surface microstructure. The original RSP was characterized by a densely packed surface that was smooth yet uneven, showcasing a schistose (flake-like) appearance. In contrast, RSP-AB8 featured a porous structure with a notably rough surface, while RSP-D101 was distinguished by its extensive filamentous network and a more relaxed structural composition. The surface of RSP-D4020 revealed a combination of sheet-like and fibrous structures, adorned with thin, debris-like fragments. RSP-HPD100’s morphology was comparatively smoother, punctuated with porous fragments. Furthermore, RSP-S8’s surface presented a mix of rough fragments and irregular filaments, interspersed with small pores. These SEM results highlighted the significant impact of ultrasound-assisted decolorization on the surface morphology of RSP.

### 3.4. In Vitro Hypoglycemic Activity of RSP before and after Decoloration

#### 3.4.1. Inhibitory Effect on α-Glucosidase

**Figure 8:**
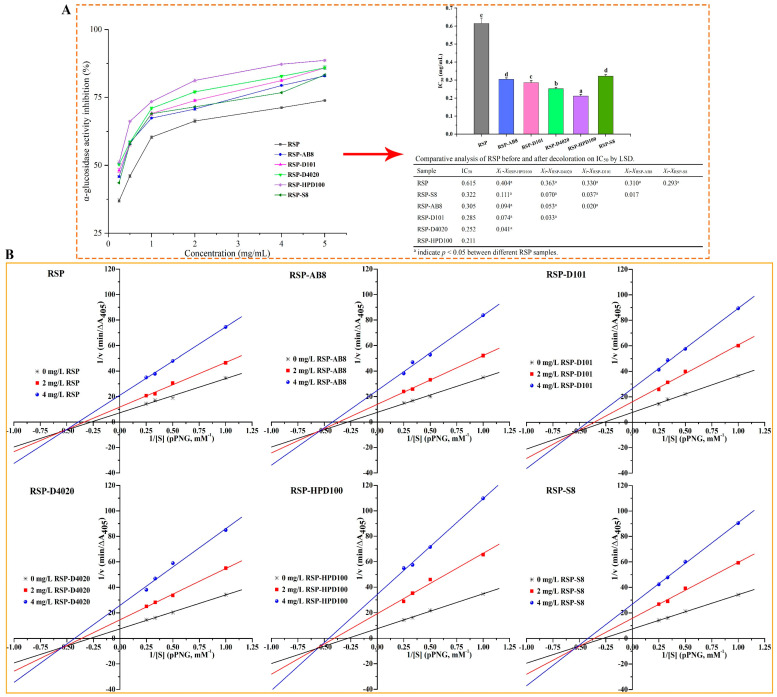
The inhibitory effects of RSPs on α-glucosidase (**A**); The double-reciprocal Lineweaver–Burk plots of the effects of RSPs on α-glucosidase (**B**). Values for IC_50_ with no letters in common are significantly different (*p* < 0.05).

Slowing down the breakdown of complex carbohydrates in the diet by inhibiting α-glucosidase enzymes is a strategic approach to prevent spikes in blood sugar levels following meals [47]. Figure 8A displays the inhibitory effects of both the original and decolored RSP at various concentrations on α-glucosidase activity. All tested RSP variants demonstrated a concentration-dependent reduction in the α-glucosidase effect. Specifically, when the concentration reached 5.0 mg/mL, the inhibition activities of RSP and its decolored counterparts RSP-AB8, RSP-D101, RSP-D4020, RSP-HPD100, and RSP-S8 were recorded at 73.88%, 82.91%, 85.82%, 85.89%, 88.63%, and 83.27%, respectively. When assessing their efficiency via the IC_50_ values, a trend emerged showcasing the following variance in inhibitory capacity: RSP (0.615 mg/mL) was surpassed by RSP-S8 (0.322 mg/mL), RSP-AB8 (0.305 mg/mL), RSP-D101 (0.285 mg/mL), RSP-D4020 (0.252 mg/mL), and RSP-HPD100 (0.211 mg/mL), sequentially. This enhancement in inhibitory activity through USMRA treatment suggested that the choice of resin significantly impacted the efficacy of RSPs against α-glucosidase, with RSP-HPD100 outperforming its counterparts. The mechanism behind this inhibition lies in the ability of polysaccharides’ hydroxyl (–OH) and carboxylic (C–OOH) groups to establish strong hydrogen bonds with the α-glucosidase, disrupting its activity [48]. Additionally, factors like molecular weight and conformation of these polysaccharides contribute to their inhibitory prowess [49]. The chemical composition and structural analysis of the RSP variants indicated that RSP-HPD100’s notably high efficacy in impeding α-glucosidase is likely due to its comparatively lower molecular weight and high uronic acid content, alongside more protruding chains that unveil more active sites for interaction.

#### 3.4.2. Inhibition Kinetics Analysis

The analysis of enzyme kinetics using the double-reciprocal Lineweaver–Burk plots, along with the associated kinetic parameters, is presented in Figure 8B and Table 4. These plots for both the original and decolored RSP converged within the third quadrant. This pattern signified a reduction in the *K_m_* and *V_max_* values for all RSP variants upon their introduction into the reaction system, accompanied by an elevation in the reciprocal of Michaelis constant (X-intercept). These findings indicated that all RSP samples facilitated a mixed-type inhibition of α-glucosidase. This mechanism of action implies that the substrate and the RSPs engage with separate, distinct active sites on the α-glucosidase enzyme. Consequently, RSPs do not compete directly with the substrate for binding but instead interact with alternative sites on the enzyme. This interaction alters the enzyme’s conformation, affecting its ability to bind to the substrate efficiently or catalyze the reaction, thereby leading to an overall reduction in enzyme activity [50]. These findings aligned with outcomes from our preceding research [17], further validating the mixed-type inhibition effect of RSPs on enzyme (α-glucosidase) activity.

Determining the dissociation constant (*K_i_*) between an enzyme and its inhibitor involves analyzing the Lineweaver–Burk plot to find the X-intercept absolute value, indicating the effect of inhibitor concentration on the plot’s slope. The dissociation constant (*K_ii_*) for the enzyme–substrate–inhibitor complex is acquired similarly, but the Y-intercept of the Lineweaver–Burk plot is considered in relation to the inhibitor’s concentration [51,52]. Analyses in Figure S4 and Table 4 revealed that all RSPs exhibited *K_i_* values greater than their *K_ii_* values, suggesting a stronger binding affinity for the α-glucosidase-PNPG complexes than for the α-glucosidase alone. Lower *K_i_* and *K_ii_* values generally indicate more robust interactions within both the inhibitor–enzyme–substrate and inhibitor-enzyme complexes [53]. Consequently, RSP-HPD100, exhibiting the lowest *K_i_* and *K_ii_* values among the RSPs, showcased the strongest binding affinity to α-glucosidase. This was corroborated by its superior inhibitory performance, as reflected in the lowest IC_50_ value, further underscoring RSP-HPD100’s potent inhibition capability. In light of these findings, HPD100 resin was selected as the optimum resin for the decolorization of RSP.

## 4. Conclusions

The present study delved into the efficacy of ultrasound-assisted decoloration of seedless chestnut rose fruit polysaccharides employing different macroporous resins. Among the resins evaluated, AB-8, D101, D4020, HPD100, and S8 stood out for their superior decoloration efficiency, polysaccharide recovery, and selectivity coefficient. The study also revealed that the choice of decolorization resin has a significant impact on the attributes of RSP, altering factors such as carbohydrate and uronic acid levels, monosaccharide profile, molecular weight and particle size distribution, as well as surface texture. These changes, in turn, influenced the RSP’s in vitro hypoglycemic effects. Notably, the use of different resins did not modify the infrared spectral characteristics, thermal resilience, or the crystallinity of RSP. Particularly, RSP decolorized with HPD100 resin, which had the highest uronic acid content and the smallest particle size and molecular weight, showcased the most potent inhibitory effect on α-glucosidase through a mixed inhibition mechanism. The use of HPD100 resin in the ultrasound-assisted static adsorption decolorization process might better maintain the macromolecular structure of RSP while boosting its α-glucosidase inhibitory potential. In essence, this research offered a validated decolorization technique for RSP and laid the groundwork for its potential application in health foods and as an α-glucosidase inhibitor, providing an essential experimental basis for future studies. RSP can be incorporated into foods like bread, cookies, or snacks as functional ingredients that provide health benefits beyond basic nutrition.

Ultrasonication, recognized for its simplicity and eco-friendliness, has found applications across a range of food processing and related sectors. Its role in the decolorization of polysaccharides through the modification of macroporous resins is a relatively new but growing area of interest. This study contributes to this growing area by exploring the modification of macroporous resins aimed at refining the purification process of RSP. The findings advocate for the use of ultrasonication to modify macroporous resins, which not only improves the decoloration efficiency of RSP but also amplifies its inhibitory effects on α-glucosidase. However, the transition from laboratory to commercial scale necessitates further research. This step is crucial for realizing the full potential of ultrasonication in the widespread and commercial decolorization of polysaccharides.

## Figures and Tables

**Table 1:** Physical and chemical properties of different macroporous resins used in the present study.

Resin	Structure	Mode of Adsorption	Appearance	Surface Area (m^2^/g)	Wet True Density (g/mL)	Sized Bead Content after Grinding (%)	Particle Size (mm)
AB-8	Cross-linked polystyrene	Non-ionic weak-polar	Milky white opaque spherical particles	480–520	1.00–1.10	≥95	0.3–1.25
ADS-17	Cross-linked polystyrene	Non-ionic hydrogen bond	White opaque spherical particles	90–150	1.03–1.07	≥90	0.3–1.25
D101	Cross-linked polystyrene	Non-ionic non-polar	Milky white opaque spherical particles	550–600	1.10–1.18	≥90	0.3–1.25
D201	Cross-linked polystyrene	Anionic-polar	Milky white opaque spherical particles	500–550	1.06–1.10	≥90	0.315–1.25
D301-G	Cross-linked polystyrene	Non-ionic weak-polar	Canary yellow opaque spherical particles	500–550	1.03–1.07	≥95	0.315–1.25
D4020	Cross-linked polystyrene	Non-ionic non-polar	White opaque spherical particles	540–580	1.01–1.05	≥90	0.3–1.25
DA201	Cross-linked polystyrene	Anionic-polar	White opaque spherical particles	500–550	1.03–1.07	≥90	0.3–1.25
HP-20	Cross-linked polystyrene	Non-ionic non-polar	White opaque spherical particles	500–600	1.03–1.07	≥90	0.3–1.25
HPD100	Cross-linked polystyrene	Non-ionic non-polar	White opaque spherical particles	650–700	1.03–1.07	≥90	0.3–1.25
HPD500	Cross-linked polystyrene	Anionic-polar	White opaque spherical particles	500–550	1.03–1.07	≥90	0.3–1.25
HPD722	Cross-linked polystyrene	Non-ionic weak-polar	Milky white opaque spherical particles	480–520	1.05–1.09	≥90	0.3–1.25
HPD826	Cross-linked polystyrene	Non-ionic hydrogen bond	White opaque spherical particles	500–600	1.03–1.07	≥90	0.3–1.25
NKA-9	Cross-linked polystyrene	Anionic-polar	Light yellow opaque spherical particles	500–550	1.03–1.07	≥90	0.3–1.25
S-8	Cross-linked polystyrene	Anionic-polar	Milky white opaque spherical particles	100–120	1.03–1.07	≥90	0.3–1.25

**Table 2:** Chemical composition, sugar composition, and molecular weight distribution of RSPs before and after decoloration by different resins.

	RSP	RSP-AB8	RSP-D101	RSR-D4020	RSP-HPD100	RSP-S8
Chemical composition (%, *w*/*w*)						
Total sugar	61.80 ± 2.62 ^a^	68.41 ± 2.82 ^b^	72.60 ± 1.18 ^c^	77.82 ± 4.90 ^d^	72.73 ± 5.67 ^c^	73.41 ± 3.92 ^c^
Protein	2.04 ± 0.07 ^a^	2.24 ± 0.09 ^a^	2.32 ± 0.09 ^a^	2.20 ± 0.07 ^a^	2.25 ± 0.09 ^a^	2.22 ± 0.08 ^a^
Uronic acid	14.26 ± 1.28 ^a^	18.66 ± 1.42 ^b^	22.05 ± 2.79 ^c^	22.39 ± 1.28 ^c^	26.51 ± 1.95 ^d^	19.08 ± 1.07 ^b^
Monosaccharide composition (molar ratio, %)						
Fuc	0.53	0.31	0.29	0.25	0.22	0.23
Rha	2.63	3.09	3.38	1.89	4.07	3.41
Ara	9.42	9.21	9.86	7.29	11.81	11.47
Gal	38.16	29.34	36.96	29.06	39.15	42.50
Glc	23.95	30.97	11.76	25.82	6.84	15.73
Xyl	3.22	1.42	1.72	1.42	2.03	1.90
Man	1.64	0.84	0.75	0.78	0.69	0.73
GalA	20.45	24.82	35.28	33.49	35.19	24.03
Molecular weight distribution						
Mw (kDa)	307.54 ± 2.31 ^a^	341.06 ± 17.96 ^b^	338.19 ± 22.28 ^b^	326.52 ± 6.37 ^a^	323.84 ± 7.58 ^a^	361.88 ± 5.75 ^c^
Mn (kDa)	40.70 ± 0.40 ^a^	58.19 ± 1.35 ^c^	49.86 ± 1.03 ^b^	57.45 ± 1.22 ^c^	42.33 ± 0.07 ^a^	46.45 ± 0.18 ^b^
Mw/Mn	7.56 ± 0.21 ^c^	5.86 ± 0.26 ^a^	6.79 ± 0.59 ^b^	5.68 ± 0.05 ^a^	7.65 ± 0.19 ^c^	7.79 ± 0.15 ^c^

Means with different letters within a row differ significantly (*p* < 0.05).

**Table 3:** Comparison of thermogravimetric stabilities of RSPs before and after decoloration by different resins.

Sample	Stage 1	Stage 2				Stage 3		Total Mass Loss (%)	*T*_50_ (°C)
	Mass Loss (%)	Start Temperature (°C)	Maximum Decomposition Rate (%/min)	*T*_max_ (°C)	Mass Loss (%)	Start Temperature (°C)	Mass Loss (%)		
RSP	9.0 ± 0.3 ^b^	129.4 ± 4.3 ^a^	4.72 ± 0.24 ^a^	224.4 ± 5.3 ^a^	60.4 ± 2.1 ^a^	574.4 ± 6.9 ^a^	4.4 ± 0.2 ^bc^	73.8 ± 2.1 ^a^	296.3 ± 5.7 ^a^
RSP-AB8	7.9 ± 0.4 ^c^	125.5 ± 3.9 ^b^	4.44 ± 0.39 ^a^	215.5 ± 4.2 ^b^	58.5 ± 2.6 ^ab^	575.4 ± 6.1 ^a^	4.9 ± 0.1 ^b^	71.3 ± 0.8 ^a^	295.9 ± 3.6 ^a^
RSP-D101	10.0 ± 0.4 ^a^	129.1 ± 5.2 ^a^	3.78 ± 0.37 ^b^	219.1 ± 6.0 ^a^	59.4 ± 2.6 ^a^	574.4 ± 4.5 ^a^	4.0 ± 0.2 ^c^	73.4 ± 1.3 ^a^	295.1 ± 5.0 ^a^
RSP-D4020	10.6 ± 0.5 ^a^	124.2 ± 2.8 ^b^	3.92 ± 0.42 ^b^	214.2 ± 5.1 ^b^	55.9 ± 1.4 ^b^	574.2 ± 5.9 ^a^	7.3 ± 0.4 ^a^	73.8 ± 1.6 ^a^	294.9 ± 4.5 ^a^
RSP-HPD100	9.2 ± 0.3 ^b^	130.6 ± 5.4 ^a^	4.55 ± 0.26 ^a^	220.6 ± 7.1 ^a^	58.1 ± 2.0 ^ab^	575.6 ± 5.1 ^a^	5.2 ± 0.3 ^b^	73.3 ± 2.2 ^a^	295.0 ± 3.3 ^a^
RSP-S8	9.8 ± 0.2 ^ab^	131.1 ± 4.0 ^a^	4.49 ± 0.34 ^a^	212.7 ± 4.9 ^b^	56.9 ± 2.8 ^b^	574.7 ± 4.3 ^a^	5.6 ± 0.2 ^b^	72.3 ± 0.9 ^a^	296.7 ± 4.2 ^a^

Values are expressed as mean ± SD (n = 3). Means with different letters within a column differ significantly (*p* < 0.05).

**Table 4:** Kinetic parameters of *α*-glucosidase inhibition in the presence of RSP before and after decoloration by different resins.

Sample	Concentration (mg/mL)	Inhibition Type	*K_m_* (mM)	*V*_max_ (∆A405/min)	*K_i_* (mg/mL)	*K_ii_* (mg/mL)	*K_i_/K_ii_*
RSP	0	Mixed inhibition	3.726	0.134	3.710	1.965	1.888
	3		2.991	0.0853			
	5		2.556	0.0477			
RSP-AB8	0	Mixed inhibition	3.601	0.132	3.257	1.577	2.065
	3		2.751	0.0720			
	5		2.368	0.0403			
RSP-D4020	0	Mixed inhibition	3.607	0.134	2.832	1.439	1.968
	3		2.773	0.0633			
	5		2.382	0.0372			
RSP-S8	0	Mixed inhibition	3.743	0.130	3.329	1.568	2.123
	3		2.775	0.0623			
	5		2.378	0.0378			
RSP-HPD100	0	Mixed inhibition	3.646	0.134	2.174	1.036	2.098
	3		2.467	0.0521			
	5		2.195	0.0292			
RSP-D101	0	Mixed inhibition	3.599	0.135	3.036	1.459	2.081
	3		2.769	0.0689			
	5		2.338	0.0388			

## Data Availability

The original contributions presented in the study are included in the article/Supplementary Materials and further inquiries can be directed to the corresponding author.

## Supplementary Materials

The following supporting information can be downloaded at: https://www.mdpi.com/article/10.3390/foods13091349/s1, Figure S1: Ion chromatograms of monosaccharide standards and hydrolysates of RSP before and after decoloration with different macroporous resins; Figure S2: The XRD spectra of RSP before and after decoloration with different macroporous resins; Figure S3: TG and DTG curves of RSP before and after decoloration with different macroporous resins; Figure S4: The slope of Lineweaver-Burk plot versus concentration for RSPs (A); The Y-intercept of Lineweaver-Burk plot versus concentration for RSPs (B).
