# Supplementary material for: Ultrasonic-Assisted Decoloration of Polysaccharides from Seedless Chestnut Rose (Rosa sterilis) Fruit: Insight into the Impact of Different Macroporous Resins on Its Structural Characterization and In Vitro Hypoglycemic Activity
Source: Foods. 2024 Apr 27;13(9):1349. doi: 10.3390/foods13091349 (PMC11083239; doi:10.3390/foods13091349)
Supplement: Supplementary file 1 [file foods-13-01349-s001.zip › foods-2972414-supplementary.pdf]

Supplemental material

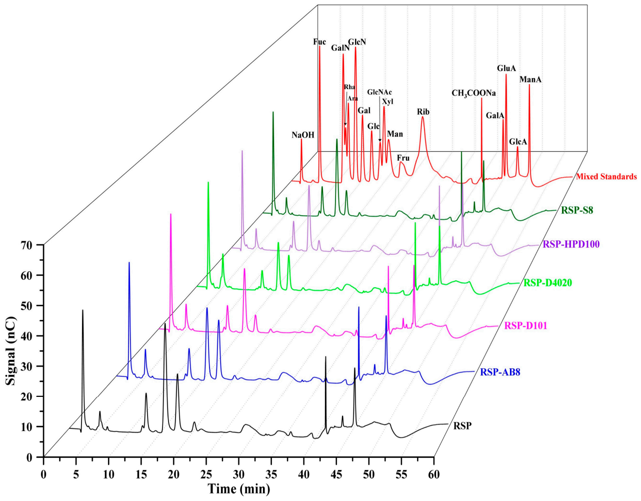

Figure S1. Ion chromatograms of monosaccharide standards and hydrolysates of RSP before and after decoloration with different macroporous resins.

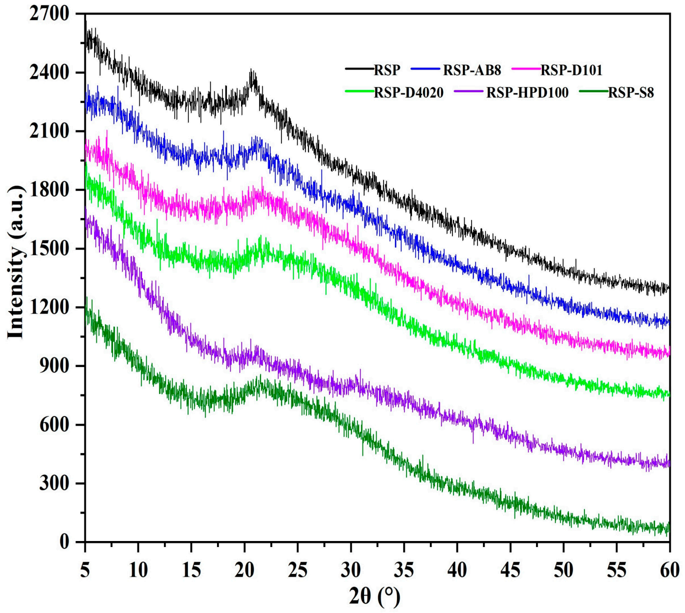

Figure S2. The XRD spectra of RSP before and after decoloration with different macroporous resins.

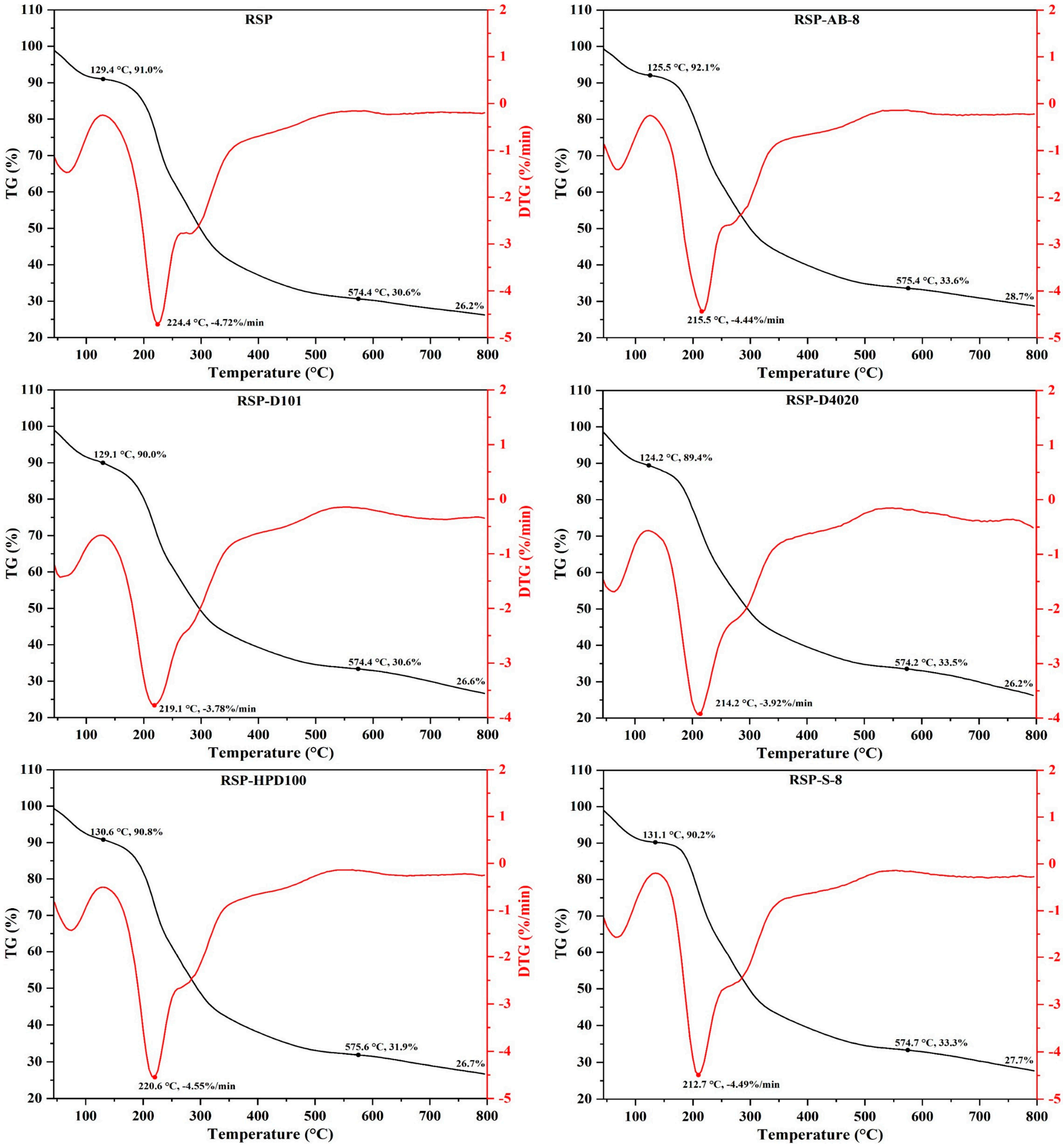

Figure S3. TG and DTG curves of RSP before and after decoloration with different macroporous resins.

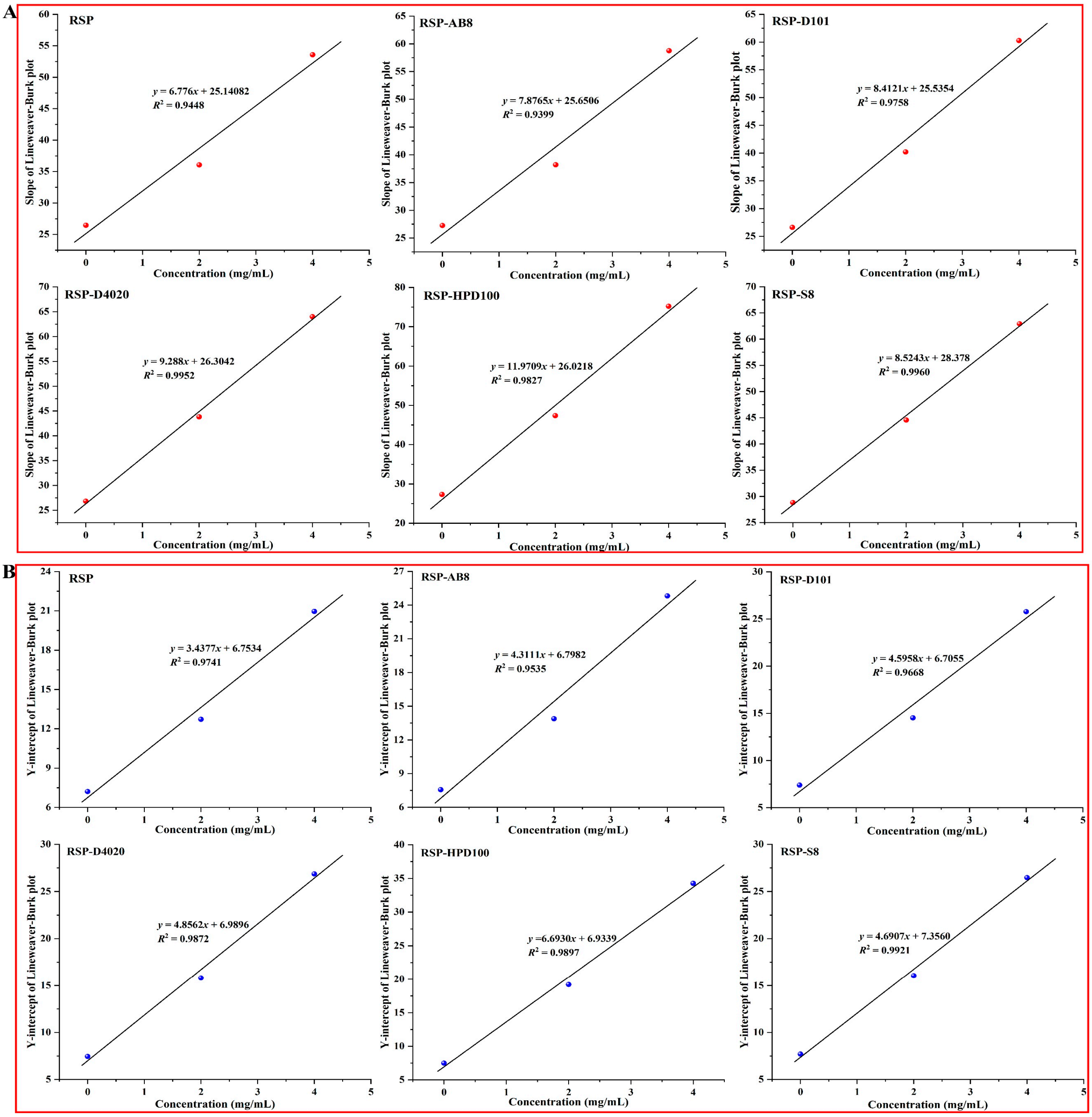

FigureS4. The slope of Lineweaver-Burk plot versus concentration for RSPs (A); The Y-intercept of Lineweaver-Burk plot versus concentration for RSPs (B).
